# Non-recombinant display of the B subunit of the heat labile toxin of *Escherichia coli* on wild type and mutant spores of *Bacillus subtilis*

**DOI:** 10.1186/1475-2859-12-98

**Published:** 2013-10-29

**Authors:** Rachele Isticato, Teja Sirec, Lucia Treppiccione, Francesco Maurano, Maurilio De Felice, Mauro Rossi, Ezio Ricca

**Affiliations:** 1 Department of Biology, Federico II University, Naples, Italy; 2 Institute of Food Sciences, C.N.R, Avellino, Italy

## Abstract

**Background:**

Mucosal infections are a major global health problem and it is generally accepted that mucosal vaccination strategies, able to block infection at their entry site, would be preferable with respect to other prevention approaches. However, there are still relatively few mucosal vaccines available, mainly because of the lack of efficient delivery systems and of mucosal adjuvants. Recombinant bacterial spores displaying a heterologous antigen have been shown to induce protective immune responses and, therefore, proposed as a mucosal delivery system. A non-recombinant approach has been recently developed and tested to display antigens and enzymes.

**Results:**

We report that the binding subunit of the heat-labile toxin (LTB) of *Escherichia coli* efficiently adsorbed on the surface of *Bacillus subtilis* spores. When nasally administered to groups of mice, spore-adsorbed LTB was able to induce a specific immune response with the production of serum IgG, fecal sIgA and of IFN-γ in spleen and mesenteric lymph nodes (MLN) of the immunized animals. Dot blotting experiments showed that the non-recombinant approach was more efficient than the recombinant system in displaying LTB and that the efficiency of display could be further increased by using mutant spores with an altered surface. In addition, immunofluorescence microscopy experiments showed that only when displayed on the spore surface by the non-recombinant approach LTB was found in its native, pentameric form.

**Conclusion:**

Our results indicate that non-recombinant spores displaying LTB pentamers can be administered by the nasal route to induce a Th1-biased, specific immune response. Mutant spores with an altered coat are more efficient than wild type spores in adsorbing the antigen, allowing the use of a reduced number of spores in immunization procedures. Efficiency of display, ability to display the native form of the antigen and to induce a specific immune response propose this non-recombinant delivery system as a powerful mucosal vaccine delivery approach.

## Background

Several vaccination strategies based on the development of microbial and viral systems to deliver molecules with antigenic properties have been proposed and recently reviewed [1-4]. In this context, bacterial endospores have also been considered to display heterologous antigens on their surface [5,6]. Endospores are produced by Gram-positive microorganisms mainly belonging to the *Bacillus* and *Clostridium* genera and including more than 1,000 species [6]. The endospore (spore), a quiescent cellular type produced in response to harsh environments, can survive in its dormant state for long periods, resisting to a vast range of stresses such as high temperature, dehydration, absence of nutrients and presence of toxic chemicals. When the environmental conditions ameliorate, the spore germinates originating a vegetative cell able to grow and sporulate [7]. The ability of the spore to survive non-physiological conditions is, in part, due to its surface structures: the coat, formed by at least seventy different proteins (Cot proteins) organized in an inner and an outer part [7] and the crust, a recently discovered outermost layer of the spore [8].

A number of reasons support the use of the spore as a vaccine delivery system. The remarkable and well documented resistance of spores to various environmental and toxic effects [6,9] ensures high stability of the display system. Proteins to be displayed on the spore are produced in the mother cell compartment of the sporangium and are assembled around the forming spore without the need to be translocated across a membrane, thus eliminating the size constrains of cell-based display systems [5,6,9]. The safety record of several endospore-forming species [10], makes spores of those species ideal candidates as vehicles to deliver molecules to mucosal surfaces.

The strategy to obtain the spore surface display of heterologous proteins is based on the construction of gene fusions between the gene coding for a selected spore surface protein (carrier) and the heterologous DNA coding for the protein to be displayed [5]. By this approach a variety of heterologous proteins have been displayed and recombinant spores proposed as vaccine vehicles [11-13], as biocatalysts [9], or as a bioremediation tool [14]. This strategy, based on the genetic engineering of the host, produces recombinant organisms to be used as a live biotechnological tool.

The release of live recombinant organisms into nature raises concerns over the use and clearance of genetically modified microorganisms and is a major drawback of all microbe-based display systems [15]. To overcome this obstacle, a non-recombinant approach to use spores as a display system has been recently proposed and model proteins efficiently exposed. The mammalian NADPH-cytochrome P450 reductase [16], the *Escherichia coli* phytase [17], the beta-galactosidase of *Alicyclobaccilus acidocaldaricus*[18] and a collections of antigens (TTFC of *Clostridium tetani*, PA of *Bacillus anthracis*, Cpa of *Clostridium perfringens* and glutathione S transferase of *Shistosomas japonica*) [19] were all adsorbed to spores and shown to either retain their specific activity [16-18] or induce specific and protective immune responses in mucosally immunised mice [19]. Spore adsorption resulted to be more efficient when the pH of the binding buffer was acidic (pH 4) and less efficient or totally inhibited at pH values of 7 or 10 [18,19]. A combination of electrostatic and hydrophobic interactions between spores and antigens were suggested to drive the adsorption, that was shown to be not dependent on specific spore coat components but rather on the negatively charged and hydrophobic surface of the spore [19]. However, at least in the case of the beta-galactosidase of *A. acidocaldaricus*, the electrostatic forces were not found essential in the adsorption to the spore surface [18]. The same study also showed that the interaction with the spore protected the enzyme from exposure to conditions of acidic pH and high temperatures and that mutants with severely altered spore surface interacted more efficiently than isogenic wild type spores with the beta-galactosidase [18].

In the present work we used a well-characterized antigen, the binding subunit of the heat-labile toxin (LTB) of *E. coli*[20] to study spore adsorption. Since LTB has been previously displayed on the spore surface by the recombinant approach [11] it was possible to compare the efficiency of display of the two approaches. Here we first show that LTB can be adsorbed to *B. subtilis* spores and that the adsorbed antigen induces a specific immune response in mucosally immunized mice. Then, we show that LTB is displayed more efficiently by the non-recombinant than by the recombinant approach and that mutant spores are more efficient than isogenic wild type spores. Finally, we show that only by the non-recombinant approach LTB is displayed in its active pentameric form.

## Results and discussion

### LTB of *E. coli* adsorbs to *B. subtilis* spores

**Figure 1:**
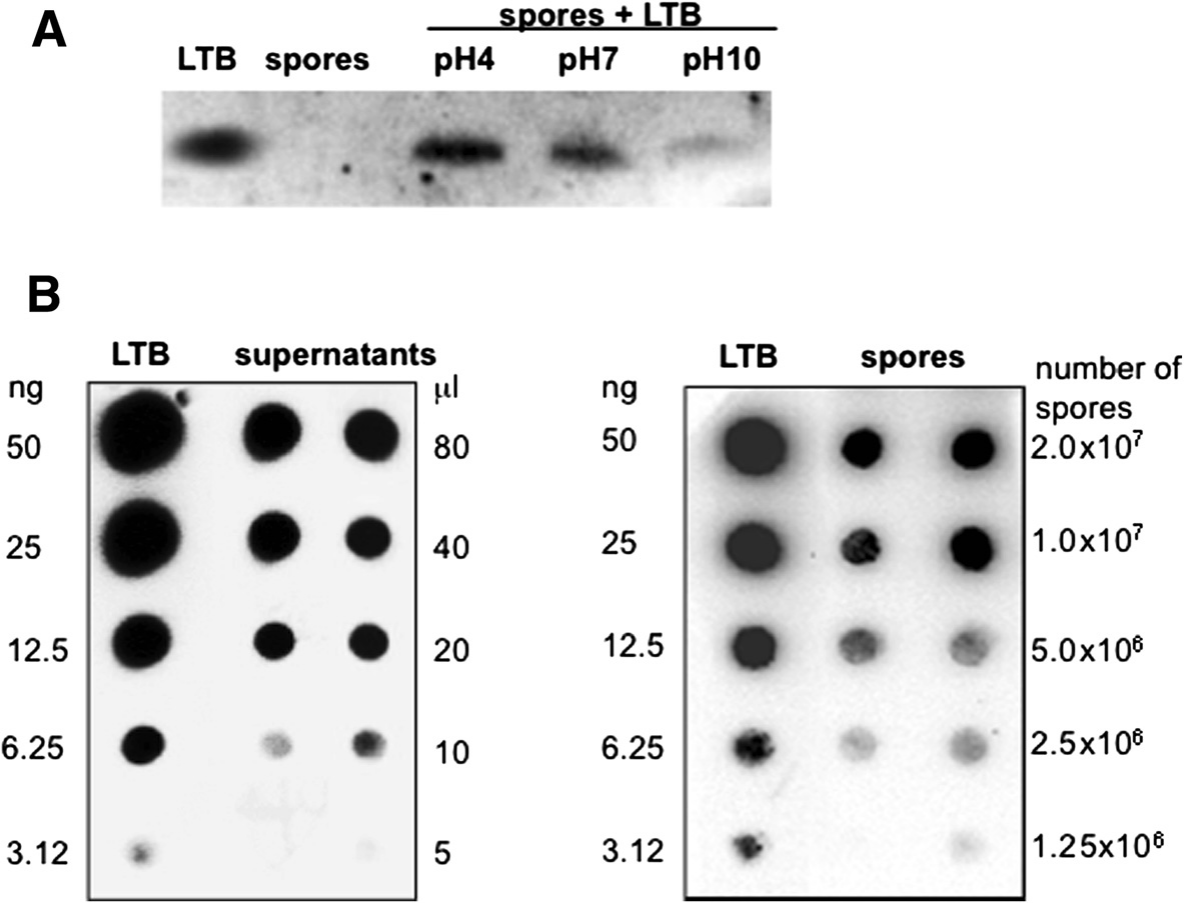
**Visualization of spore adsorption by western and dot blotting experiments. (A)** Upon adsorption with purified LTB at various pH conditions, spore surface proteins were extracted by SDS-DTT treatment, fractionated on SDS-PAGE and analyzed by western blot. **(B)** The adsorption mixture (200 μl) was fractionated by centrifugation and different amounts of pellet (spores) and supernatant (free LTB) fractions were analyzed by dot blot. Serial dilutions of purified LTB were used as a standard. Immuno-reactions in **A** and **B** were performed with anti-LTB antibody [11] and anti-rabbit secondary antibody conjugated with the horseradish peroxidase.

Aliquots (2.0 μg) of LTB, over-expressed in *E. coli* and purified by affinity chromatography columns (Methods), were incubated in 200 μl of PBS buffer with 2.0 × 10^9^ spores of the *B. subtilis* strain PY79 [21], purified by renographin gradient [22]. After one hour of incubation at 25°C spores were collected by centrifugation and surface proteins extracted by SDS-DTT treatment [18]. Proteins were then analyzed by western blot with anti-LTB antibody [11] and LTB was found among the proteins extracted from the spore surface (Figure 1A). As previously reported for other antigens [19] and an enzyme [18], the adsorption was more efficient when the binding reaction was performed at pH 4.0 than at pH 7.0 and was almost abolished at pH 10.0 (Figure 1A).

In order to assess the amount of LTB adsorbed on the spore surface, we fractionated by centrifugation the adsorption reaction mixture obtaining a pellet with the spores and LTB bound to them and a supernatant with unbound, free LTB. Both fractions were analyzed by dot blot with anti-LTB antibody (Figure 1B) and the intensity of the various spots quantified by a densitometric analysis. In our experimental conditions (pH 4.0, 2.0 × 10^9^ spores and 2.0 μg of purified LTB in 200 μl) about 2% of LTB was left free in the supernatant (Table 1) while about 80% of it was detected in the pellet fraction (Table 2) and therefore bound to spore. The remaining 18% of LTB used in the adsorption reaction was either lost in the washing steps of the dot blot experiment or degraded.

**Table 1:** Densitometric analysis of dot blot experiments with the supernatants of the adsorption reaction with wild type spores

**LTB source**	**Amount of sample used**	**Density (OD/mm**^**2**^**)**	**Amount of LTB** **(ng)**	**Amount of LTB μg in 200 μl (% total)**
Purified LTB	12.50 ng	20.125	NA	NA
	6.25 ng	8.753	NA	NA
	3.12 ng	3.696	NA	NA
Free LTB	40 μl	13.211	8.8	0.04 (2.2)
	20 μl	6.689	3.9	0.03 (1.9)
	10 μl	3.507	2.2	0.04 (2.2)

**Table 2:** Densitometric analysis of dot blot experiments with the pellets of the adsorption reaction with wild type spores

**LTB source**	**Amount of sample used**	**Density (OD/mm**^**2**^**)**	**Amount of LTB (ng)**	**Amount of LTB (μg/** **2.0 × 10**^**9 **^**spore) (% total)**
Purified LTB	25.00 ng	12.259	NA	NA
	12.50 ng	6.689	NA	NA
	6.25 ng	3.507	NA	NA
Bound LTB	1.0 × 10^7^	5.219	9.6	1.92 (96)
	5.0 × 10^6^	2.756	4.3	1.72 (86)
	2.5 × 10^6^	1.450	1.5	1.20 (60)

### Spore-adsorbed LTB induces a specific immune response in nasally immunized mice

**Figure 2:**
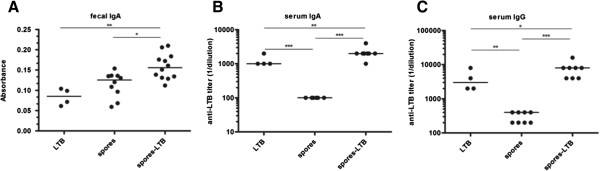
**Antibody production in mice immunized with spore-adsorbed LTB.** BALB/c mice were treated with antigen alone (n=4) (LTB), spores alone (n=10) (spores) or LTB-adsorbed spores (n=12) (spores-LTB). **(A)** Anti-LTB IgA detected in fecal pellet at the end of treatment; absorbance values are shown. Anti-LTB IgA **(B)** and IgG **(C)** levels detected in mice serum; reciprocal endpoint titers are shown. Bars represent median values. Results are representative of two independent experiments. *, *P* < 0.05; **, *P* < 0.01, ***, *P* < 0.001.

In a previous study, the gene coding for LTB was fused to the gene coding for the spore coat protein CotC and the recombinant strain of *B. subtilis* shown to display an average of 9.6 × 10^-5^ pg of CotC-LTB per spore [11]. Nine doses of 1.0 × 10^10^ recombinant spores (corresponding to 0.55 μg of LTB/dose for a total of 4.95 μg of LTB) were used to mucosally immunize groups of mice and the LTB-specific immune response monitored [11]. In particular, the production of low levels of LTB-specific serum IgG and fecal sIgA was observed [11]. To analyze whether spore-adsorbed LTB molecules were also able to induce an immune response, groups of mice were nasally immunized with purified LTB (2.0 μg/dose), spores alone (2.0 × 10^9^/dose) or spore-adsorbed LTB (2.0 × 10^9^ spores adsorbed with 2.0 μg of LTB/dose). Animals were dosed once a week for 8 weeks and were sacrificed for analysis one week after the last dose. As calculated in the previous paragraph, 2.0 × 10^9^ spores adsorbed with 2.0 μg of LTB displayed about 1.6 μg of LTB (80% of the total LTB), therefore, eight doses of spores carried 12.8 μg of LTB, about 2.5-fold more than that delivered by the recombinant approach but using a 5-fold lower number of spores (2.0 × 10^9^ instead of 1.0 × 10^10^ per dose). A statistically higher production of fecal sIgA, indicative of a mucosal immune response, was observed in mice immunized with spore-adsorbed LTB than in mice immunized with spores alone (negative control), whereas purified LTB was unable to induce sIgA at the tested dose (Figure 2A). The analysis of serum antibodies showed that both anti-LTB IgA (Figure 2B) and IgG (Figure 2C) titers were significantly increased following administration of spore adsorbed LTB in comparison to antigen alone. We speculate that the ability of nasally administered spore-adsorbed LTB to induce a stronger immune response than purified LTB could be related to an increased antigen uptake by immune competent cells or, alternatively, to a reduced antigen degradation, as previously shown for the beta-galactosidase of *A. acidocaldaricus*[18]. Further experiments are required to fully address this issue.

**Figure 3:**
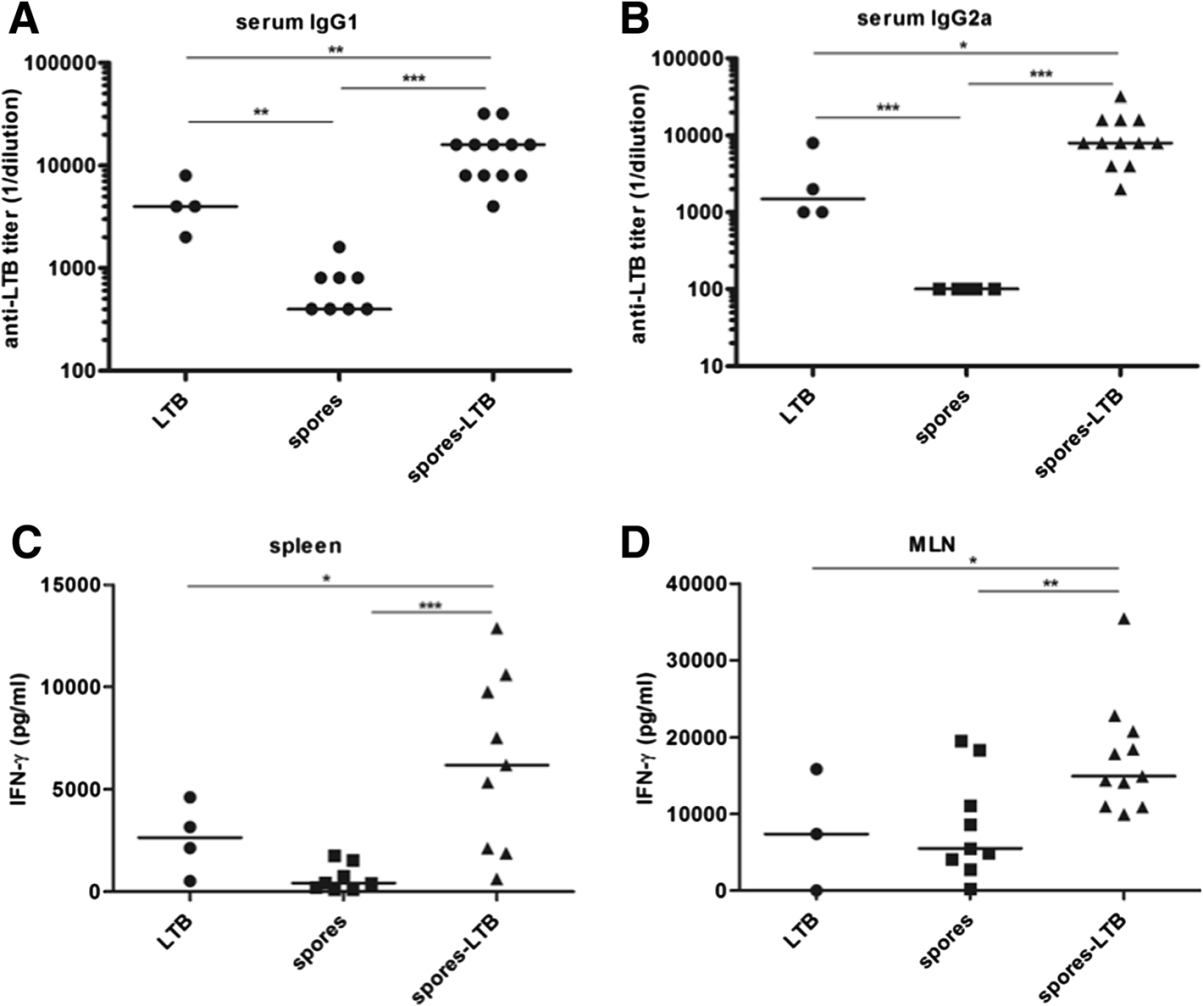
**Characterization of the immune response.** A Th1-like immunity was induced in BALB/c mice following administration of LTB-adsorbed spores. Anti-LTB IgG1 **(A)** and IgG2a **(B)** levels detected in mice; reciprocal endpoint titers are shown. Antigen-specific IFN-γ levels secreted *in vitro* from spleen **(C)** and MLN cells **(D)**; data are reported after subtracting the cytokine values detected in the absence of antigen stimulation. Bars represent median values. Results are representative of two independent experiments. *, *P* < 0.05; **, *P* < 0.01; ***, *P* < 0.001.

The phenotype of the induced immune response was then examined. The analysis of IgG subclasses did not show a preferential increase of IgG1 or IgG2a subtypes (Figure 3AB). Spleen and mesenteric lymph nodes (MLN) of immunized animals were analyzed for LTB-specific production of IL-4 and interferon-γ (IFN-γ). While IL-4 was not produced at detectable levels (not shown), IFN-γ was produced by both spleen and MLN cells of mice immunized with spore-adsorbed LTB at levels statistically higher than by those immunized with purified LTB (Figure 3CD). Although a comparison between our data and those previously obtained with recombinant spores displaying LTB [11] is not possible for the different immunization route (oral vs. nasal) and the different amounts of antigen administered (4.95 vs. 12.8 μg), our results confirm the previously reported humoral response. Moreover, the statistically significant production of IFN-γ and the lack of detectable levels of LTB-specific IL-4 of immunized animals suggest that the nasal administration of spore-adsorbed LTB promotes a significant cellular (Th1-biased) immune response in both systemic and mucosal compartments. Induction of a Th-1 type of immune response by antigens carried by spores is in agreement with previous observations deriving from experiments with spores displaying different antigens [19,23].

### Increased efficiency of LTB display

**Figure 4:**
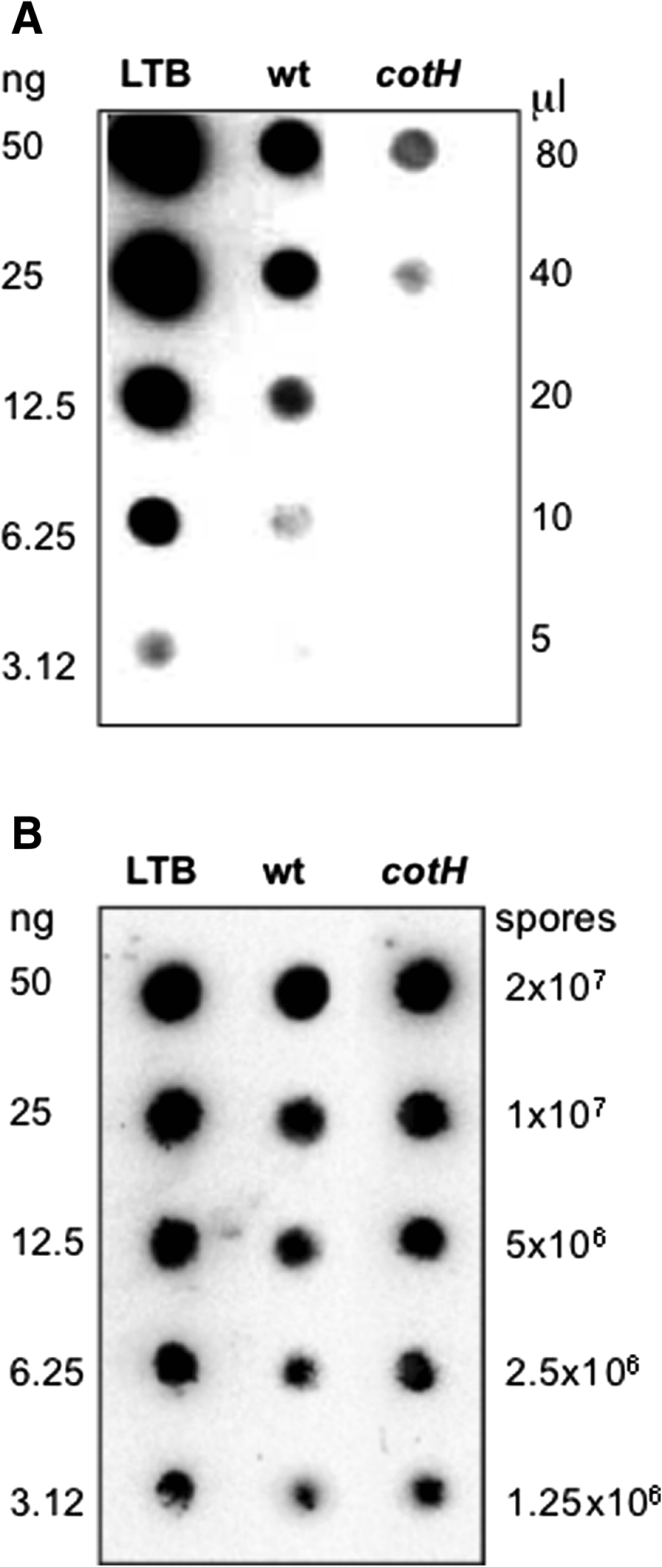
**Efficiency of LTB adsorption on wild type and mutant spores.** Upon adsorption with 2.0 μg of purified LTB the reaction mixture was fractionated by centrifugation. Supernatant **(A)** and pellet **(B)** fractions were serially diluted and analyzed by dot blot. Serial dilutions of purified LTB were used as a standard. Immuno-reactions in **A** and **B** were performed with anti-LTB antibody [11] and anti-rabbit secondary antibody conjugated with the horseradish peroxidase.

The non-recombinant display system was more efficient than the recombinant approach and allowed the delivery of a high amount of antigen with a low number of spores. To verify whether it was possible to further increase the amount of LTB adsorbed to spores we tested a collection of mutants of *B. subtilis* with an altered spore surface. In agreement with previous data obtained with various antigens [19] and an enzyme [18], LTB adsorption was similar in wild type spores (PY79) and in isogenic null mutants lacking *cotA, cotB, cotC, cotD* or *cotG* (not shown). However, it has also been reported that the spore adsorption of the beta-galactosidase of *A. acidocaldaricus* was significantly increased in *cotH* null mutant spores that have a strongly altered coat [18]. We then analyzed the adsorption of LTB to *cotH* mutant spores. After one hour of incubation at 25°C we fractionated by centrifugation the adsorption reaction as described above, and performed dot blot experiments with anti-LTB antibody on both supernatant (free LTB) and pellet (spore-bound LTB) fractions (Figure 4). A densitometric analysis of the various spots indicated that the amount of LTB not bound to spores was much lower in the mutant than in the isogenic wild type strain (Figure 4A and Table 3), while spore-bound LTB was much higher in the mutant than in the wild type (Figure 4B and Table 4). Therefore, results of Figure 4 and Tables 3 and 4 indicate that mutant spores with a strongly altered surface are more efficient than wild type spores in adsorbing LTB and confirm previous data on the adsorption of the beta-galactosidase of *A. acidocaldaricus*[18].

**Table 3:** **Densitometric analysis of dot blot experiments with the supernatants of the adsorption reaction with wild type and *cotH *** **mutant spores**

**LTB source**	**Amount of sample used**	**Density (OD/mm**^**2**^**)**	**Amount of LTB (ng)**	**Amount of LTB μg in 200 μl (% total)**
**Purified LTB**	12.50 ng	25.125	NA	NA
	6.25 ng	10.875	NA	NA
	3.12 ng	4.621	NA	NA
**wt**	40 μl	16.517	9.2	0.04 (2.0)
	20 μl	8.361	4.1	0.04 (2.1)
	10 μl	4.383	1.9	0.03 (1.9)
***cotH***	80 μl	7.522	3.8	0.009 (0.4)
	40 μl	2.987	1.2	0.006 (0.3)

**Table 4:** **Densitometric analysis of dot blot experiments with the pellets of the adsorption reaction with wild type and *cotH *** **mutant spores**

**LTB source**	**Amount of sample used**	**Density (OD/mm**^**2**^**)**	**Amount of LTB (ng)**	**Amount of LTB (μg/2.0 x 10**^**9 **^**spore) (% total)**
**Purified LTB**	25.0 ng	75.553	NA	NA
	12.5 ng	40.385	NA	NA
	6.25 ng	22.113	NA	NA
**wt**	1 x 10^7^	38.631	8.2	1.64 (82)
	5 x 10^6^	15.227	3.5	1.41 (70)
	2.5 x 10^6^	9.238	1.6	1.29 (65)
***cotH***	5 x 10^6^	18.527	4.7	1.90 (95)
	2.5 x 10^6^	7.857	2.2	1.836 (91)
	1.25 x 10^6^	3.864	1.1	1.888 (94)

**Figure 5:**
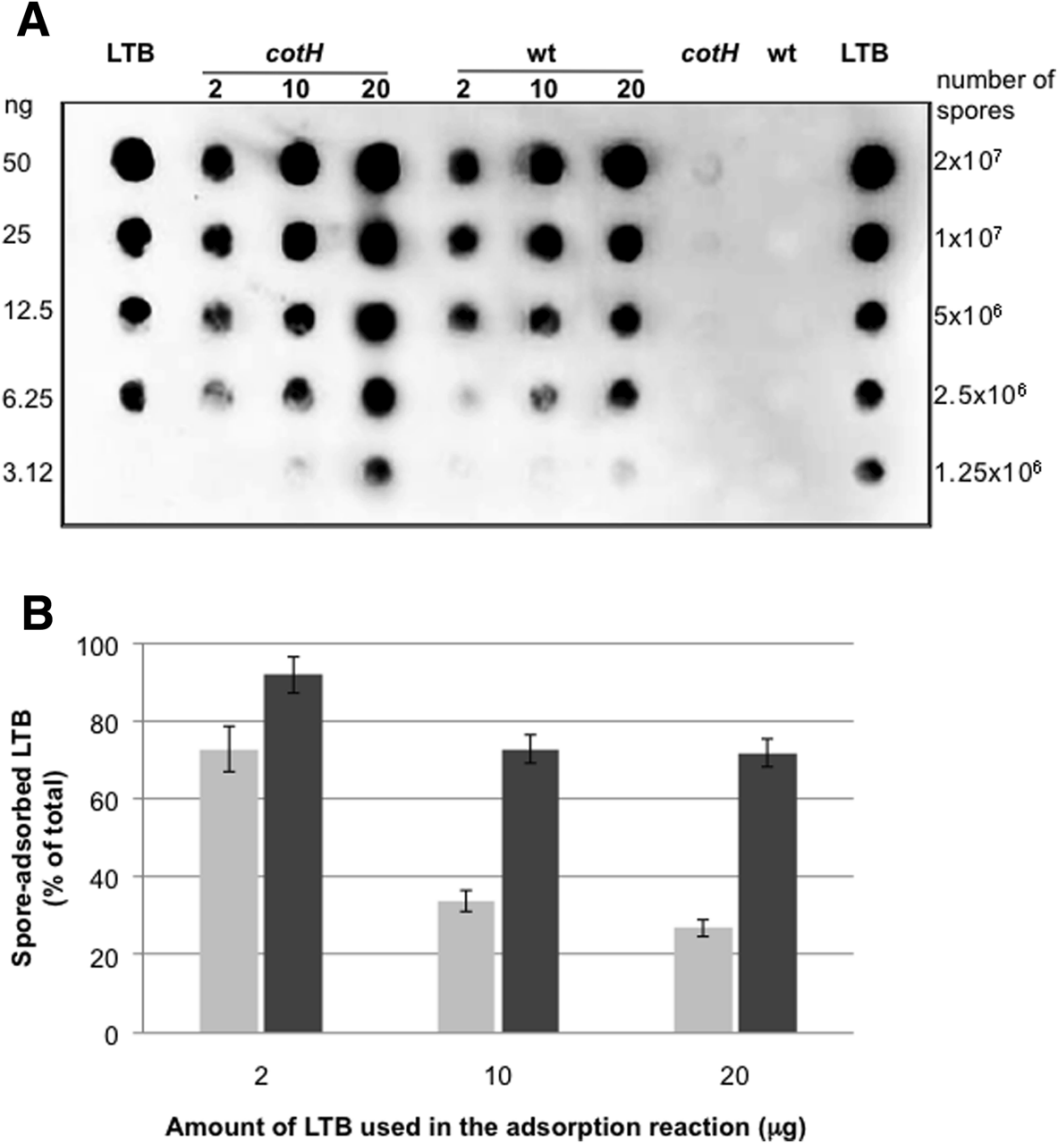
**Saturation of LTB adsorption. (A)** The adsorption reactions were performed with 2.0 × 10^9^ spores of the wild type and *cotH* mutant strain and with 2.0, 10.0 and 20.0 μg of purified LTB. Serial dilutions of purified LTB were used as a standard. Wild type and mutant spores not adsorbed with LTB were used as negative controls. Immuno-reactions were performed with anti-LTB antibody [10] and anti-rabbit secondary antibody conjugated with the horseradish peroxidase. **(B)** Percentage of LTB bound to the spore surface of wild type (gray bars) or *cotH* mutant (black bars) spores. The amount of LTB used in the adsorption reaction was considered as 100%. Error bars represent the standard deviation of each experiments.

Both wild type and *cotH* mutant spores were then tested for adsorption with increasing amounts of LTB. Dot blot (Figure 5A) and densitometric analysis (Table 5) of the obtained spots indicated that the amount of spore-bound LTB increased when high amounts of purified LTB were used in the adsorption reaction. With wild type spores about 1.5, 3.4 and 5.5 μg of LTB was displayed after adsorption with 2.0, 10.0 or 20.0 μg of purified LTB, respectively (Table 5). With *cotH* mutant spores, the efficiency of adsorption is higher than with wild type spores at all tested LTB concentrations and 1.8, 7.3 and 14.3 μg of LTB were displayed on mutant spores using for the adsorption reaction 2.0, 10.0 and 20.0 μg of purified LTB, respectively (Table 5). Densitometric data of Table 5 are summarized in Figure 5B and show that with wild type spores (grey bars) the efficiency of LTB display decreased from about 70% to 30% when high amounts of purified LTB were used in the adsorption reaction. With *cotH* mutant spores (black bars in Figure 5) the efficiency of LTB only slightly decreased and remained around 70% when 10.0 and 20.0 μg of purified LTB were used in the adsorption reaction.

**Table 5:** **Densitometric analysis of dot blot experiments with the pellets of the adsorption reaction with wild type and *cotH *** **mutant spores and increasing amounts of LTB**

**LTB source**	**Amount of sample used**	**Density (OD/mm**^**2**^**)**	**Amount of LTB (ng)**	**Amount of LTB μg/2 x 10**^**9 **^**spore μl** **(total)**
**Purified LTB**	25.0 ng	122.592	NA	NA
	12.5 ng	66.385	NA	NA
	6.25 ng	35.721	NA	NA
**2 μg**				
**wt**	1 × 10^7^	36.331	7.2	1.50 (75)
	5 × 10^6^	22.387	3.8	1.52 (75)
	2.5 × 10^6^	14.418	1.2	1.45 (72)
***cotH***	5 × 10^6^	30.527	4.2	1.92 (96)
	2.5 × 10^6^	19.157	2.3	1.86 (93)
	1.25 × 10^6^	9.564	1.3	1.79 (89)
**10 μg**				
**wt**	1 × 10^7^	98.331	19.7	3.94 (39)
	5 × 10^6^	51.387	8.7	3.48 (35)
	2.5 × 10^6^	28.418	3.6	2.88 (28)
***cotH***	1 × 10^7^	142.37	32	6.4 (64)
	5 × 10^6^	97.285	19	7.6 (76)
	2.5 × 10^6^	53.644	10	8.0 (80)
**20 μg**				
**wt**	5 × 10^6^	66.778	12.8	5.1 (25)
	25 × 10^6^	42.422	7.5	6.0 (30)
***cotH***	5 × 10^6^	159.87	35.2	14.3 (71)
	2.5 × 10^6^	87.56	17.34	13.8 (70)
	1.25 × 10^6^	42.422	9.83	15.0 (75)

### Spore-adsorbed LTB is in its active pentameric form

**Figure 6:**
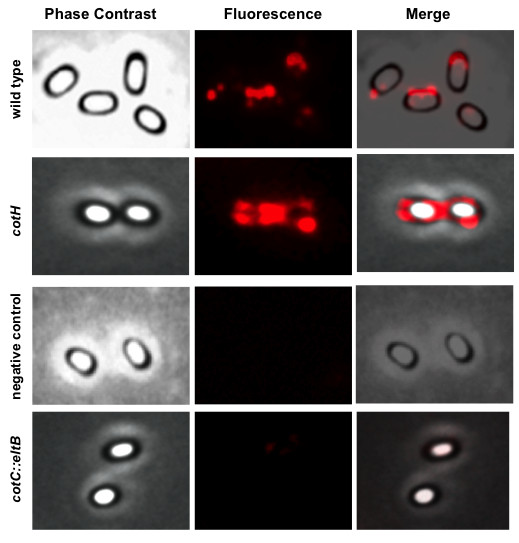
**Immunofluorescence microscopy with anti-GM1 antibody.** Wild type, *cotH* mutant and recombinant *cotC::eltB* spores were adsorbed with 10.0 μg of LTB, treated with GM1 and reacted with anti-GM1 antibody and Texas Red conjugated secondary antibody. Spores adsorbed with LTB but not treated with GM1 were also reacted with GM1 antibody (negative control). A representative microscopy field for each strain is observed by phase contrast and immunofluorescence. Merged panels of phase contrast and immunofluorescence are shown. Exposure times were typically in the range of 50–200 ms.

The heat-labile toxin of *E. coli* is similar to the cholera toxin and is formed by an enzymatically active A subunit and a pentameric non-toxic B subunit (LTB). Only in its pentameric form LTB can bind its receptor, the ganglioside GM1, allowing entry of the A subunit into the host eukaryotic cell. LTB alone is not toxic but able to induce a potent mucosal immunogenic activity. In addition, LTB can exert a mucosal adjuvant activity for coadministered unrelated antigens [24]. Although the mechanism of the adjuvant effects of LTB is not known, such activity strictly depends on LTB ability to bind to its cell receptor GM1, and therefore on the pentameric association of five B subunits [24]. When LTB is expressed in a heterologous bacterium fused to an anchor protein, pentamer formation is impaired [25]. In order to assess whether LTB pentamers were formed when LTB was displayed by the non-recombinant system on the spore surface, an immunofluorescence microscopy analysis with anti-GM1 antibody was performed. Wild type and *cotH* mutant spores of *B. subtilis* were adsorbed with 10 μg of purified LTB as described above, reacted with GM1 (*Merck*) and used for immunofluorescence analysis with anti-GM1 antibody. As shown in Figure 6, spores of both strains were fluorescent indicating that GM1 was able to bind the spore surface and, therefore, that LTB pentamers were present on the spore surface. As expected for the high efficiency of LTB adsorption, *cotH* mutant spores were more fluorescent than the wild type spores (Figure 6). When wild type or mutant spores were adsorbed with LTB but not treated with GM1, no fluorescence was observed after reaction with anti-GM1 antibody (negative control in Figure 6), indicating the specificity of the fluorescent signals observed when GM1 was used. The recombinant strain carrying a *cotC::eltB* gene fusion and displaying the CotC-LTB fusion on the spore surface [11] was also used for immunofluorescence analysis with anti-GM1 antibody. As shown in Figure 6 no fluorescence was observed around the recombinant spores, indicating that no pentamers were present.

## Conclusions

In this study we analyzed the adsorption of the B subunit of the heat-labile toxin (LTB) of *Escherichia coli* to spores of *Bacillus subtilis.* Although the recombinant and non-recombinant display of antigens and enzymes on *B. subtilis* spores has been reported previously [5,6,9,11-19], our work highlights several new information that significantly improve the potential applications of the spore-based display system. First of all, the non-recombinant spore display approach based on surface absorption turned out to be significantly more efficient than the recombinant approach, the latter being based on the construction of gene fusions between DNA coding for a spore coat protein (carrier) and for LTB (passenger). An average of 9.6 × 10^-5^ pg of CotC-LTB per spore was previously shown to be displayed by a strain carrying a *cotC::eltB* gene fusion [11]. We report here that using 20.0 μg of purified LTB in the adsorption reaction, up to 2.5 × 10^-3^ pg of LTB per spore are displayed on wild type spores. This about 25-fold increase of displayed LTB becomes even larger by using mutant spores altered in the spore surface. Indeed, with *cotH* null mutant spores, up to 7.0 × 10^-3^ pg of LTB per spore were displayed, resulting in a 2.5- and 70-fold increase with respect to wild type spores and to the recombinant approach, respectively. Mutant spores lacking CotH have been previously characterized [26,27]. In addition to CotH they lack at least nine other coat proteins (CotH-dependent coat proteins) [28], and as a consequence have strong defects in both inner and outer coat [27]. These alterations reduce the total negative charge of the spore [18] and we speculate that they could also affect other physicochemical properties of the spore relevant for adsorption. An increased efficiency of display is particularly relevant when the system is aimed at delivering an antigen to a mucosal surface, since it allows to reduce the number of spores needed to induce an immune response. Nine doses of 1.0 × 10^10^ recombinant spores induce a specific immune response in mice [11]. However, scaling-up that number to immunize humans would be extremely difficult or even not realistic, making essential to develop more efficient display systems.

A second main result of this work is that LTB forms pentamers on the spore surface. Consistently with a previous report where LTB was displayed on the surface of *Streptococcus gordonii* cells [25] also when expressed on the spore surface as a fusion protein with CotC, LTB does not form pentamers (Figure 6). We show here that the native, pentameric form of LTB was observed only when purified LTB was adsorbed to spores. This aspect is crucial since the immunostimulatory activity of LTB largely depends on its ability to bind to its cell receptor, most commonly the ganglioside GM1, which in turn requires the association of B subunits to form a donut-shaped pentamer via noncovalent associations. Pentamer formation and interaction with the receptor result in enhanced targeting and access to MHC compartments [29] with the consequent increased activation of antigen presenting cells and T cells [30].

In conclusion, our work shows that by a non-recombinant approach LTB can be efficiently displayed in its native pentameric form on the spore surface and that spore-displayed LTB molecules induce a Th1-biased immune response in mice immunized by the nasal route. These data, together with the well established robustness and stability of the spore and the safety record of spores of several species, propose the spore as a very promising platform to display molecules to be presented to the human mucosal surfaces.

## Methods

### Bacterial strains and transformation

The *B. subtilis* PY79 strain [21] was used as a wild type. Strain ER220 (*cotH* null mutant) [26] and all other mutant strains of *B. subtilis* used in this study were isogenic derivatives of PY79 and have all been described elsewhere [18]. Isolation of plasmid DNA, restriction digestion, ligation of DNA and transformation of *E. coli* competent cells were carried out by standard methods [31].

### Purification of spores and LTB

Sporulation of wild type and recombinant strains was induced by the exhaustion method. After 30 h of growth in Difco Sporulation medium (DSM) at 37°C with vigorous shaking, spores were collected, washed three times with distilled water and purified by renografin gradient as described before [22]. Spore counts were determined by serial dilution and plate-counting.

A recombinant plasmid containing the *eltB* gene, excluding the leader peptide coding sequence, of enterotoxigenic strains of *E. coli* has been previously described [25]. A DNA fragment of 321-bp was cleaved with *Kpn*I and *Hind*III restriction enzymes and ligated into the expression vector pRSETA (*Invitrogen*), previously digested with the same enzymes. The recombinant plasmid carrying an in-frame fusion of the 5’ end of the *eltB* coding region to six histidine codons under the transcriptional control of a T7 promoter was used to transform competent cells of the *E. coli* strain BL21(DE3) (*Invitrogen*), yielding strain RH153. This strain was grown in ampicillin-supplemented (50 μg/ml) LB medium to an optical density of 0.7 at 600 nm. The T7 promoter was then induced by adding isopropyl-β D-thiogalactoside (IPTG; final concentration, 0.5 mM) to the culture, which was incubated for 4 h at 37°C [32]. The six-His-tagged LTB protein was purified under native conditions using the His-Trap column as recommended by the manufacturer (*GE Healthcare Life Science*). Purified protein was desalted using the PD10 column (*GE Healthcare Life Science*) to remove high NaCl and imidazole concentrations.

### Adsorption reaction

Purified LTB was added to a suspension of 2 × 10^9^ spores in 0,15 M PBS pH 4.0 at 25°C in a final volume of 200 μl. After 1 hour of incubation, the binding mixture was centrifuged (10 min at 13,000 g) to fractionate pellet and supernatant. The pellet was resuspended in 0,15 M PBS at pH 4.0 to a final concentration of 2 × 10^5^ spores / μl and store at 4°C for further experiments.

### Western and dot-blot analysis

2.0 × 10^8^ spores adsorbed LTB were resuspended in 20 μl of spore coat extraction buffer [22], incubated at 68°C for 1 hour to solubilize spore coat proteins and loaded onto a 12% SDS-PAGE gel. The proteins were then electrotransferred to nitrocellulose filters (*Amersham Pharmacia Biotech*) and used for Western blot analysis as previously reported [33].

A quantitative determination of the amount of LTB was obtained by dot blot experiments with specific anti-LTB antibodies analyzing serial dilutions of purified LTB, binding assay supernatant and spore adsorbed LTB. Filters were then visualized by the ECL-prime (*Amersham Pharmacia Biotech*) method and subjected to densitometric analysis by Fluor-S Multimager (*Bio-Rad*).

### GM1 binding assay

2.0 × 10^9^ spores were adsorbed with 10 μg LTB and an aliquot of 2.0 × 10^6^ reacted with 2 μg of Monosialotetrahexosyl-ganglioside (GM1)(*Merck*) in 50 μl of 1×PBS, pH 4.0 for 4 hours at 25°C. After three washes with PBS, the samples were pretreated with 1% bovine serum albumin (BSA) - 1× PBS, pH 4.0 for 30 minutes prior to incubation overnight a 4°C with the polyclonal anti-GM1 antibodies (rabbit) (*Abcam Ltd*) diluted 1:10 in PBS–1% BSA. As a control of the specificity of this technique, both spores alone and spores adsorbed with LTB were not reacted with GM1 and directly treated with anti-GM1 antibodies. After three washes, the samples were incubated with a 64-fold diluted anti-rabbit secondary antibody conjugates with Tetramethyl Rhodamine, TRITC (*Santa Cruz Biotechnology, Inc*.) and washed four times with PBS. Washed samples were resuspended in 20 μl of PBS and 10 μl analyzed using an immunofluorescence microscopy as previously described [34].

### *In vivo* treatments

Female BALB/c mice were maintained in pathogen-free conditions at the animal facility of the Institute of Food Sciences and used at the age of 8–14 weeks. The studies were approved by the National institutional review committee. Mice lightly anaesthetized with methoxuflurane were intranasally administered once/week for 8 weeks with 2.0 μg of LTB or 2 × 10^9^ LTB-adsorbed spores. Control mice received only spores. Fecal pellets were collected at the end of each week to monitor the induction of the LTB-specific immunity. Mice were then sacrificed to collect, serum samples, spleens and mesenteric lymph nodes (MLN).

### Antibody analysis

Fecal pellets were homogenized in PBS + 0.01% sodium azide (100 mg/ml) and centrifuged at 10,000 g 10 min; supernatants were recovered and stored frozen or immediately analysed.

Fecal anti-LTB IgA, serum anti-LTB IgA, IgG, IgG1 and IgG2a subclasses antibodies were assayed by ELISA. LTB was coated onto U-bottomed microtiter plates at 1.0 μg/ well overnight at 4°C. Plates were blocked with PBS containing 2% BSA. After washing with PBS containing 0.05% Tween-20 serial twofold dilutions of serum or fecal supernatants were added to individual wells and incubated for 2 hr at room temperature. The presence of antibodies was detected with peroxidase-conjugated rabbit anti-mouse IgA, IgG or isotype specific antibodies (Dako SpA, Milan, Italy) and the reaction developed with 1 mg/ml o-phenylendiamine/HCl substrate and 0.06% H_2_O_2_. Results were expressed as absorbance values after blank subtraction or as reciprocal endpoint titer of the last dilution exhibiting an O.D. ≥ 0.1 above negative controls.

### Cytokine secretion assay

MLNs and spleens were passed through a stainless-steel wire mesh to dissociate cells. Removal of erytrocytes from spleen cells was performed by using a Tris-buffered ammonium chloride solution. Cells were cultured at 2.5 × 10^6^ cells/ml in the presence/absence of LTB (10 μg/ml); after 72 hr, the supernatants were collected and analyzed for IFN-γ and IL-4 protein levels by ELISA using commercial kits.

### Statistical analysis

Differences among the various experimental groups were determined by the Kruskal-Wallis non-parametric test, followed by Dunn’s Multiple Comparison Test for post test analysis.

## Competing interests

The authors declare that they have no competing interests.

## Authors’ contribution

RI - performed western, dot blot and immunofluorescence microscopy experiments and contributed to design experiments; TS - performed western and dot blot experiments; LT - performed the immunization experiments and analyzed the immune response; FM - performed the immunization experiments and analyzed the immune response; MDF - contributed to experiment design and manuscript writing; MR - contributed to experiment design and manuscript writing; ER - contributed to experiment design and wrote most of the manuscript. All authors read and approved the final manuscript.
